# Defect-Engineered Perovskites: Atomic Scale Nature of A‑Site Vacancy-Stabilized Catalytically Active Phase

**DOI:** 10.1021/jacs.5c22657

**Published:** 2026-03-02

**Authors:** Roham Talei, Asghar Mohammadi, Thomas F. Winterstein, Christoph Malleier, Guido Schmitz, Simon Penner, Nicolas Bonmassar

**Affiliations:** † Department of Materials Physics, Institute for Materials Science, 9149 University of Stuttgart, Heisenbergstr. 3, 70569 Stuttgart, Germany; ‡ Department of Physical Chemistry, 27255 University of Innsbruck, Innrain 52c, A-6020 Innsbruck, Austria

## Abstract

Using aberration-corrected electron microscopy and spectroscopy, we reveal the atomic scale structure and catalytic function of the A-site-deficient perovskite La_0.7_Fe_0.7_Mn_0.3_O_3_, uncovering a heterogeneous defect landscape that governs its activity in reducing NO by CO. We identified a layer of La_0.7–*x*_Fe_0.7_Mn_0.3_O_3_ that is highly A-site-deficient. This layer is just two to three unit cells thick at the surfaces and the interfaces of the perovskite particles and transitions toward the bulk into stoichiometric LaFe_0.7_Mn_0.3_O_3_. These confined defect layers stabilize catalytically active sites, enabling the formation of FeO_*x*_ inclusions ranging from approximately 1  nm to several nanometers in size at the surfaces. In situ surface characterization and catalytic measurements reveal that these interfacial FeO_*x*_ nanoparticles serve as active sites during the NO reduction by CO via the Mars–van Krevelen mechanism. Our findings establish a direct relationship between the structure and properties of nanoscale A-site nonstoichiometry and redox-driven catalytic activity. This relationship offers a new design strategy for tailoring reactivity through defect engineering.

## Graphical abstract

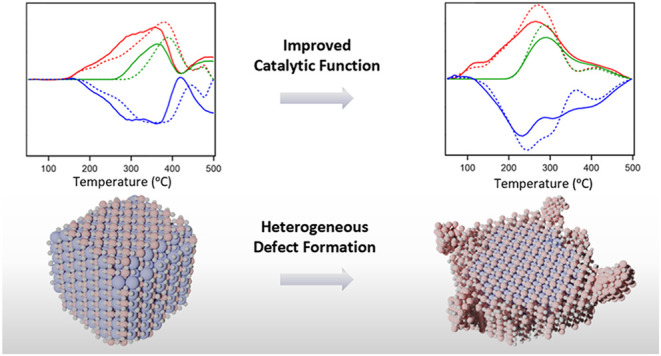

## 1 Introduction

Minimizing the use of noble metals in catalysis is essential for resource efficiency due to their scarcity,^1,2^ high cost, and environmentally damaging extraction processes.^3^ Substituting these metals with inexpensive, thermally stable oxide materials, such as perovskites,^4,5^ provides a sustainable alternative that maintains catalytic efficiency.^6^ This approach not only reduces costs, but is also a step toward more economical and environmentally friendly industrial processes. A-site-deficient perovskites represent a particularly promising replacement for noble metals, as their adjustable structures and catalytic properties make them well suited for diverse catalytic reactions. Recently, A-site deficient perovskite catalysts have been used in different fields of electrochemistry and heterogeneous catalysis,^7,8^ particularly in water oxidation and oxygen reduction,^9–11^ hydrogen evolution reaction,^12,13^ CO oxidation,^14^ ethanol steam reforming,^15^ and selective catalytic reduction/exhaust gas purification (deNO_*x*_).^16^ The common feature of these reactions is that the catalytically active site is mediated by the redox properties of the B-site cation.^17^ Hence, the surface termination of either A-site or B-site cations directly influences the catalytic activity,^18^ highlighting the importance of atomic scale insights for optimizing the catalytic performance of ABO_3_ perovskites. Accordingly, modern catalysis deliberately uses defect engineering in perovskites: A-site deficiency and aliovalent B-site substitution create oxygen vacancies and modify B-site reducibility,^19,20^ while controlled exsolution generates highly dispersed metal (or metal oxide) nanoparticles supported on the perovskite.^5,21^ Additionally, substituting A- or B-site cations in perovskites alters the catalyst’s density of states,^22,23^ and can induce oxygen vacancies, which can lead to possible adsorption sites for reactants.^24^

In our work, we use the A-site deficient and B-site substituted exhaust gas catalyst material La_0.7_Fe_0.7_Mn_0.3_O_3_ (L_0.7_FM)^16^ as a model system for defect engineering to answer key questions in heterogeneous catalysis and electrochemistry concerning the nature of defects in A-site deficient perovskites. We selected L_0.7_FM, which has the lowest La content of this material class that still exhibits a single-phase perovskite structure. It rivals state-of-the-art, perovskite-free Pd/Al_2_O_3_ materials and noble metal-containing LFM-based materials in terms of catalytic activity and N_2_ selectivity.^16^ It is noteworthy that surface termination alone cannot explain the significant La deficiency observed in these samples. Neagu et al. demonstrated that upon reduction in H_2_, the A-site deficient and substituted La_0.8_Ce_0.1_Ni_0.4_Ti_0.6_O_3_ perovskite exhibits exsolved Ni nanoparticles supported on the perovskite,^25^ similar to stoichiometric LaNiO_3_.^21^ However, despite extensive previous research and a strong recent interest in A-site deficient perovskites,^26–29^ there is, to the best of our knowledge, no report about a detailed atomic scale characterization of such perovskites and the nature of the formed defects that could explain the similarities in X-ray diffraction (XRD) of A-site deficient and non-A-site deficient perovskites reported in literature.^10,16,30,31^

Our focus is on the interfaces and surface-near areas of L_0.7_FM in order to gain insight into the origin of the off-stoichiometry and the surface segregation of B-site cations. A key question is how A-site deficiencies are distributed within the sample volume. We will clarify whether the missing A-site cations are uniformly distributed throughout the material or accumulate preferentially in certain areas. To achieve this, we utilize scanning transmission electron microscopy (STEM) including high-angle annular dark-field (HAADF) imaging and atomically resolved quantitative energy dispersive X-ray spectroscopy (EDXS) for atomistic insights into the chemical composition. Also, energy-loss near edge structure (ELNES) analysis obtained from electron energy-loss spectroscopy (EELS) data is used for valence quantification before and after catalysis. Going beyond qualitative studies, we characterize defects with atomic resolution and combine chemical quantification, electronic configuration and in situ diffuse reflectance infrared Fourier transform (DRIFT) spectroscopy with a quantification of strain at interfaces of perovskite nanoparticles and microstrain analyses by XRD. Finally, our multifaceted approach enables us to derive specific structure–property relationships by identifying the formation mechanism of the active sites. In conclusion, our work clarifies the formation of catalytically active sites, which are responsible for the improved catalytic activity compared to non-A-site deficient catalysts.

## 2 Results

### 2.1 A-site Deficiency and Heterogeneous B-site Cation Distribution

The elemental compositions of the samples, as determined by EDXS, inductively coupled plasma (ICP) analysis, and X-ray photoelectron spectroscopy (XPS) are shown in Table S1. These complementary techniques enabled both bulk and surface characterization of the materials and primarily served as evidence for the A-site deficient perovskite formation. EDX provided a rapid semiquantitative analysis of the elemental distribution across the sample, ICP offered precise quantification of the bulk elemental concentrations, and XPS enabled surface-sensitive analysis revealing La-rich surfaces. The EDX and ICP measurements are in good agreement, identifying the chemical compositions of the bulk samples and proving the differences in stoichiometry between LFM and L_0.7_FM.

In the following sections, we aim to explain the defect chemistry of the A-site deficient perovskites at the atomic scale in combination with in situ methods to derive a robust structure–property relation.

The individual perovskite nanoparticles are around 10–40 nm in size (see Figure 1a,b). The stoichiometric LFM catalyst features slightly larger particles than the A-site deficient L_0.7_FM, as confirmed by the Rietveld refinement in Table S2. ADF images reveal that both perovskite materials consist of agglomerated nanoparticles/grains within a porous structure. Visualizing the key differences between LFM and L_0.7_FM via EDXS (cf. Figure 1c,d) reveals a striking initial finding: heterogeneous Fe distribution at the nanoscale, i.e., Fe-enriched surfaces (red areas in Figure 1d) of nanoparticles and interfaces between nanoparticles. Conversely, the stoichiometric LFM catalyst (Figure 1c) does not exhibit such a heterogeneous distribution of Fe. Furthermore, quantitative EDXS measurements averaged over several agglomerated nanoparticles confirm the stoichiometry for both the LFM and the L_0.7_FM catalysts (see Figure S1g,o). The heterogeneous distribution of B-site cations in the form of nanoscale Fe-rich inclusions suggests the existence of localized redox-active environments that may directly influence the catalytic behavior. To assess this, we examined the reactivity of LFM and L_0.7_FM under NO + CO reaction conditions. Figure 1e,f show the NO conversion, NO consumption and product (CO_2_ and N_2_) formation rates during NO reduction by CO on LFM and L_0.7_FM, respectively. The promoting effect of the A-site deficiency is clearly visible in the shift of the NO reduction onset temperature from 180 °C for LFM to approximately 120 °C for L_0.7_FM. The weakly positive NO formation rates at temperatures around 100 °C indicate a nonreactive desorption of NO species from the catalyst surface. This is also confirmed by diffuse reflectance infrared Fourier transform spectroscopy (DRIFTS) results (cf. Figure 3). The early formation of CO_2_ at 100 °C, before NO consumption, could be due to Fe^2+^ on the catalyst’s surface, and is more pronounced in L_0.7_FM. This observation highlights the higher reducibility of L_0.7_FM compared to LFM, a finding that has been confirmed by previous CO and H_2_ reduction studies.^16^ This striking difference in reducibility and catalytic behavior forms the basis for our atomic scale characterization, which enables us to draw mechanistic conclusions about the catalytic behavior. (Note that N_2_O is an intermediate byproduct of the reaction and its formation profiles are shown in Figure S2, alongside conventional NO conversion data and N_2_ selectivity as a function of temperature during the first and second catalytic cycles).

**Figure 1:**
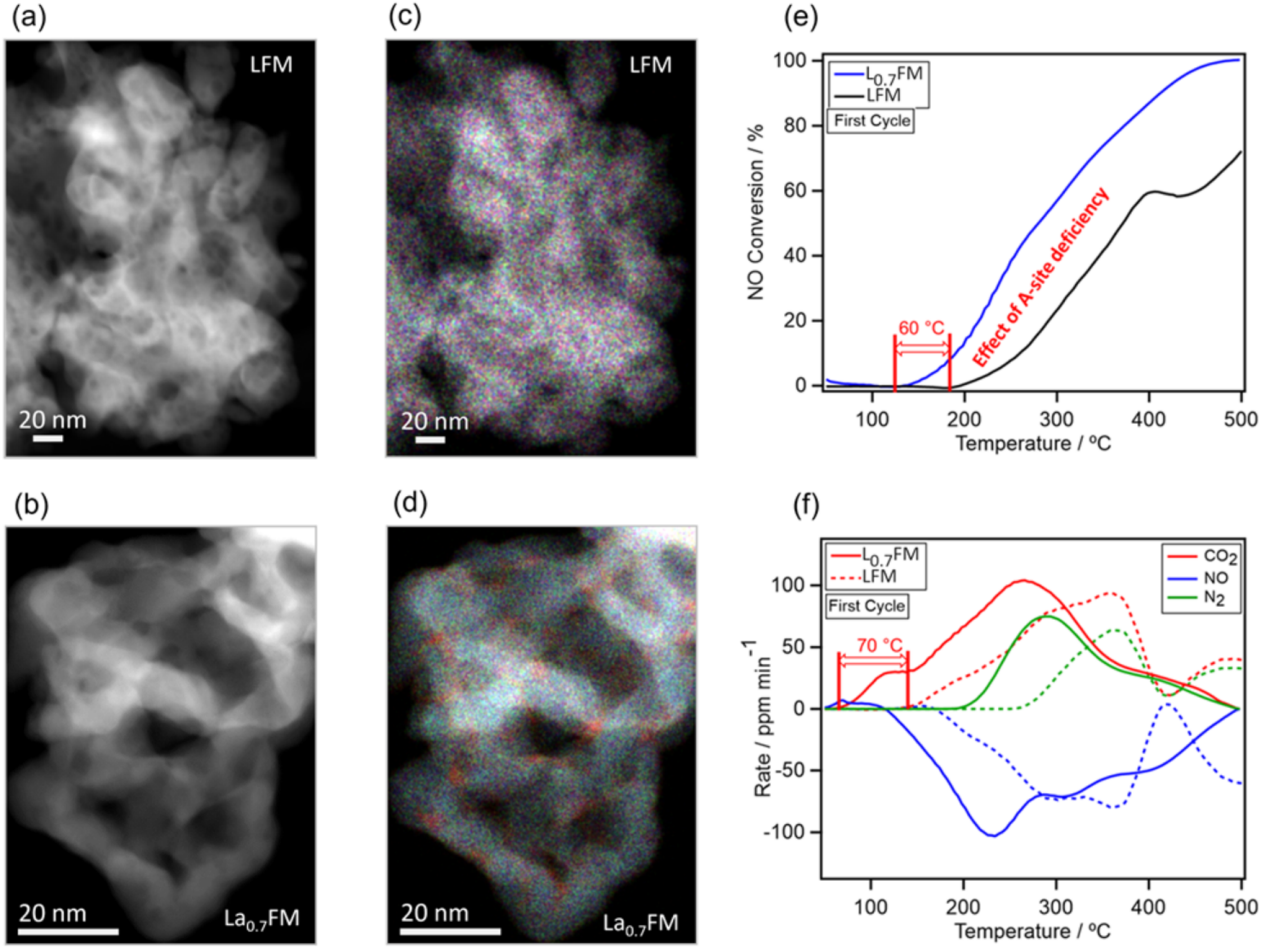
Heterogeneous B-site cation distribution in A-site deficient catalysts leading to superior catalytic performance. (a), (b) HAADF overview images of LFM and L_0.7_FM, respectively. (c) Overlay of La (blue), Fe (red) and Mn (green) EDXS signals visualizing a homogeneous distribution of elements. (d) Overlay of La (blue), Fe (red) and Mn (green) EDXS signals visualizing a heterogeneous distribution of Fe between nanoparticles and at the surface of nanoparticles. NO conversion (e) NO consumption and CO_2_/N_2_ formation rate profiles (f) during first catalytic cycle of NO reduction by CO on LFM and L_0.7_FM as a function of temperature. Note that the error margin for the two catalytic profiles lies between 2 and 3 °C. The total gas flow rate was 200 mL min^–1^ with a composition of CO/NO/He = 1:1:98 for the inlet flow. The heating ramp was 2 °C min^–1^. For these measurements 200 mg of catalyst material was used.

Figure 2a shows a Z-contrast HAADF image providing atomic scale insights into the morphology and the crystal structure of the initial L_0.7_FM catalyst before catalysis. From this overview, it can be concluded that the initial L_0.7_FM catalyst exhibits grain boundaries, as indicated by the white rectangle or the pink square, as well as numerous many other defects such as dislocations. It should be noted that the L_0.7_FM catalyst surface is covered with an atomically thin amorphous layer. This feature is also present in the stoichiometric LFM as shown in Figure S3 and could result from surface degradation induced by the environmental exposure or from contrast variations associated with hydrocarbon contamination from the isopropanol used in transmission electron microscopy (TEM) sample preparation. Quantitative chemical analysis indicates the presence of Fe-rich and La-poor regions near and inside the grain boundaries at the nanoscale, cf. line profile in Figure 2b.

**Figure 2:**
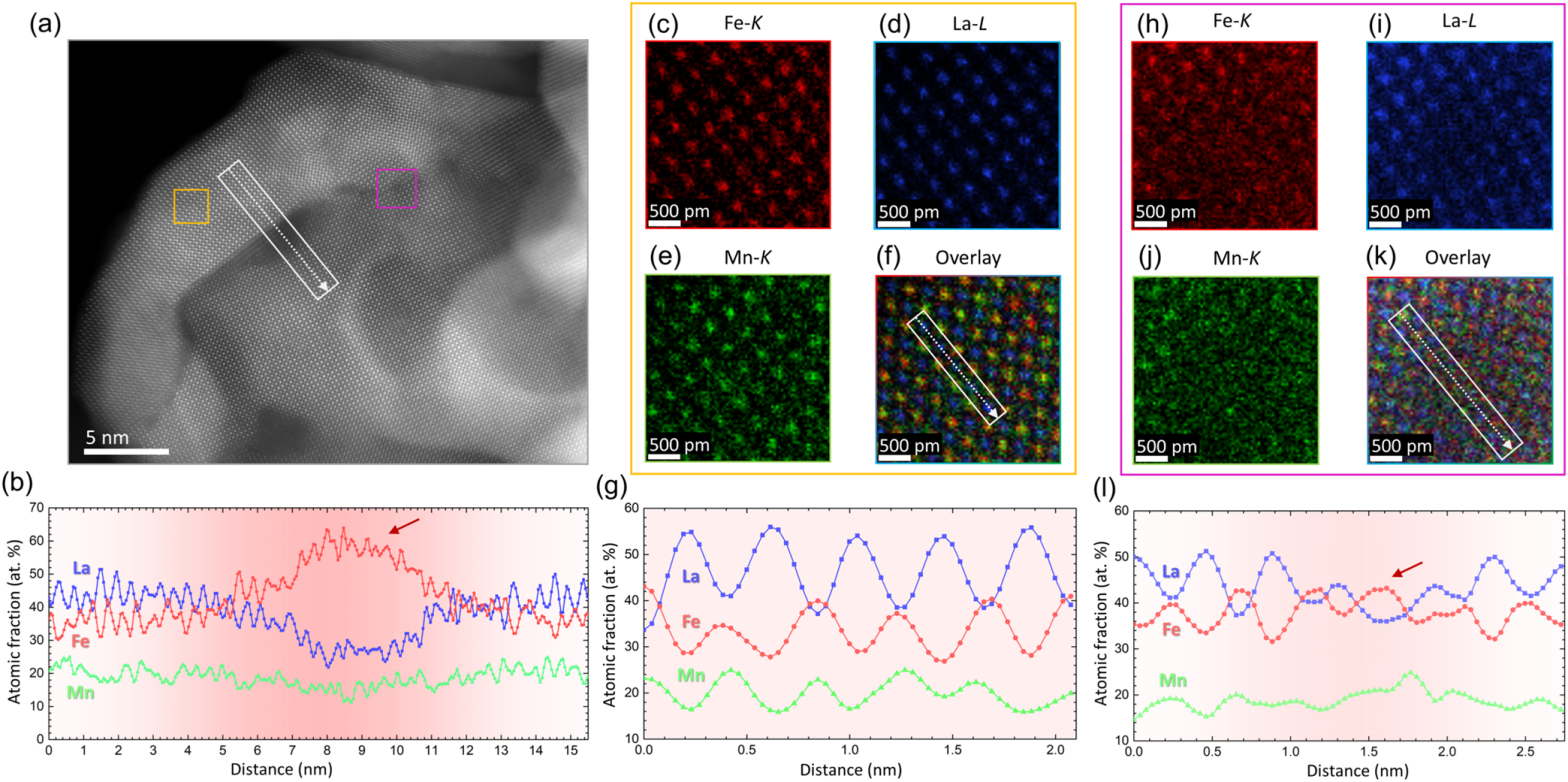
Atomic scale insights into the defects of the L_0.7_FM catalyst before catalysis. (a) HAADF overview image highlighting three distinct areas, white rectangle and two squares (orange and pink), where quantitative atomic scale EDXS measurements have been performed. The atomically resolved nanoparticles are visualized along the [100] zone axis. (b) Elemental line profile along the white rectangle of panel a highlighting the changes in atomic fraction of La (blue), Fe (red), and Mn (green) at the low angle grain boundary. Errors in panel (b) are ± 4 at. % for Fe, ± 2 at. % for Mn, and ± 3 at. % for La. (c, d, e, f) Atomically resolved elemental mappings of Fe, La, Mn, and the overlay of these three elements, respectively, show the stoichiometry in the bulk. (g) Elemental line profile in the bulk area along the white rectangle of panel f highlighting the changes in atomic fraction of La (blue squares), Fe (red circles), and Mn (green triangles). Errors in panel (g) are ± 3 at. % for Fe, ± 2 at. % for Mn, and ± 3 at. % for La. (h–k) Atomically resolved elemental mappings of Fe, La, Mn, and the overlay of these three elements, respectively, show the stoichiometry in an A-site deficient area. (l) Elemental line profile in the defective area along the white rectangle of panel k highlighting the changes in atomic fraction of La, Fe, and Mn. Errors in panel (l) are ± 4 at. % for Fe, ± 2 at. % for Mn, and ± 3 at. % for La. Red arrow points out a positions with an accumulation of Fe and a deficiency of La. Note that the sample thickness was estimated to be 15 nm.

The second key finding of this study is that the A-site deficiency manifests in a strongly La-poor La_0.7‑*x*_Fe_0.7_Mn_0.3_O_3_ perovskite that is just a few unit cells thick (five unit cells in this case) as indicated in Figure 2b. This strongly A-site deficient area is in direct proximity to a stoichiometric non-A-site deficient perovskite in the bulk as it is shown in the atomically resolved chemical mappings of Figure 2c–f and the elemental line profile in Figure 2g obtained along the white arrow shown in Figure 2f. As shown in Figure 2h–l, the A-site deficiency can also be attributed to La-poor and Fe-rich grain boundaries. Neither area has suffered severe knock-on damage from electron beam irradiation, as demonstrated by the two HAADF images in Figure S4, which were obtained after the EDX measurements. Interestingly, the quantity of Fe enrichment varies drastically when different regions of grain boundaries within the same nanoparticles are investigated, i.e., 63 ± 4 at. % in Figure 2b and 44 ± 4 at. % in Figure 2l. Based on our high-resolution chemical mapping, we conclude that A-site–deficient perovskites do not constitute a spatially homogeneous phase, but rather form a heterogeneous microstructure composed of chemically distinct domains, including interfacial “hotspots” of A-site depletion. We observe clear A-site cation depletion at grain boundaries, resulting in localized regions of A-site deficient La_0.7–*x*_Fe_0.7_Mn_0.3_O_3_. Therefore, our results suggest that the heterogeneously distributed A-site-deficient areas in direct proximity to Fe-rich nanoparticles are crucial for the understanding of the catalytic activity of these systems.

Our next aim is to clarify the catalytic behavior of the L_0.7_FM catalyst, particularly its surface state, in order to link its catalytic performance to our atomic scale observations and deduce clear structure–property relationships.

### 2.2 Characterization of Adsorbed Surface Species

In Figure 3, we determine the adsorbed gas molecules by in situ DRIFT spectra of L_0.7_FM and LFM during heating in a NO + CO reaction mixture. As the temperature increases from 25 to 100 °C, the only change observed is a decrease in the amount of chelating nitrite species, which is possibly due to nonreactive desorption (see the blue spectrum). However, significant changes are seen in the spectrum as the temperature increases to 200 °C. The amount of nitrite species decreases significantly, and new bands form in the bidentate carbonate region (1630 cm^–1^, 1605–1580 cm^–1^ and 1290 cm^–1^) on both samples.^32,33^ It is important to note that this spectral assignment is further supported by NO adsorption experiments conducted on both fully oxidized and partially reduced catalyst samples, cf. Figure S5. These results confirm that nitrate formation is favored under oxidizing conditions as indicated by Figure S5d. Conversely, under NO + CO reaction conditions, increasing the temperature is expected to induce partial reduction of the catalyst rather than oxidation. Therefore, the emergence of new bands in the 1630 cm^–1^–1300 cm^–1^ region at 200 °C is attributed to the formation of carbonate species.

**Figure 3:**
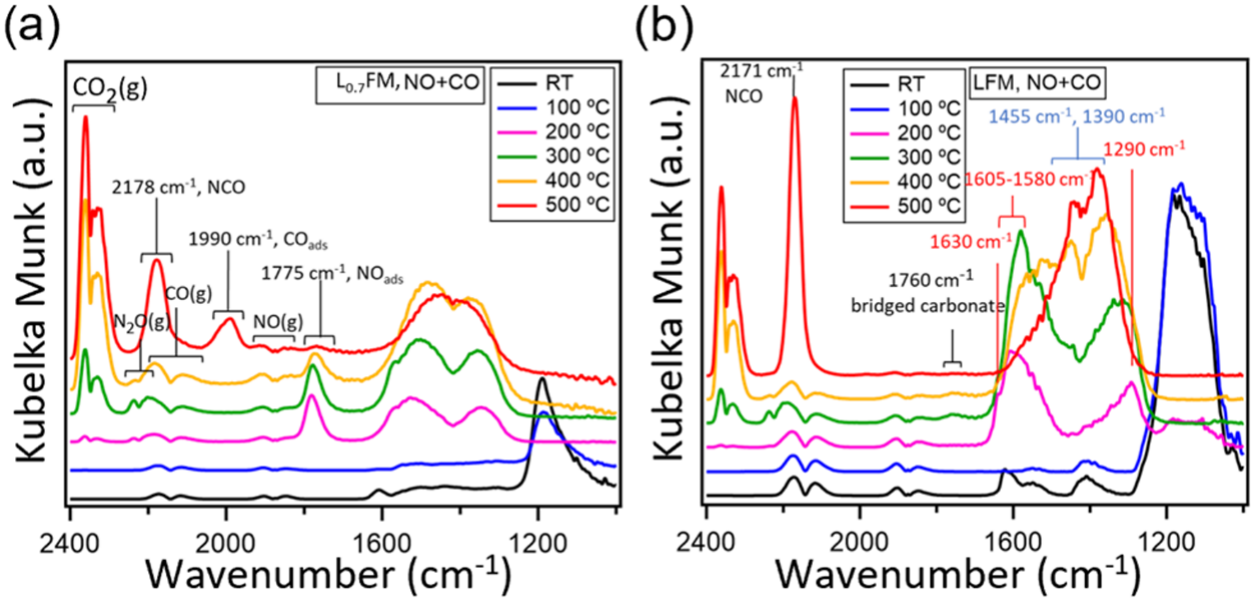
In situ DRIFT spectra of NO + CO adsorption. Adsorbed species on (a) L_0.7_FM and on (b) LFM at different temperatures in NO + CO atmosphere. The pretreatment was performed under He atmosphere. The total adsorption flow rate was 80 mL min^–1^ (CO/NO, 1:1, 5% in He). The numbers in red and blue represent bidentate and monodentate carbonate compounds, respectively.

In addition, at 200 °C the formation of gas phase CO_2_ and N_2_O is observed for both catalysts, but in the case of L_0.7_FM it is more significant confirming the higher reactivity of L_0.7_FM in accordance with catalytic measurements (Figure S2). The main difference between L_0.7_FM and LFM is the regeneration of the nitrosyl band at 1782 cm^–1^ for the L_0.7_FM sample.^34^ Based on these observations, we propose a mechanistic model for the He-treated samples under NO + CO flow: At room temperature, surface species originating from NO dominate the catalyst surface. Upon heating, these adsorbed NO-related species progressively desorb, thereby exposing active sites that can accommodate CO adsorption and enable subsequent reaction. The CO oxidation proceeds via a Mars–van Krevelen (MvK)–type mechanism, whereby lattice oxygen participates in the reaction, leading to partial surface reduction. This redox process regenerates Fe^2+^ sites, which serve as active centers for subsequent NO adsorption. Accordingly, NO species adsorbed on Fe^2+^ sites are identified as catalytically relevant intermediates within this temperature regime.

As the temperature increases to 300 °C, the intensity of gas-phase N_2_O and CO_2_ signals increase on both samples, indicating enhanced catalytic activity. On L_0.7_FM, the nitrosyl band exhibits a slight red shift to 1778 cm^–1^, while on LFM a new broad but weak band emerges around 1760 cm^–1^. Notably, the temperature-induced shift of the nitrosyl band on L_0.7_FM occurs in the opposite direction to that observed during NO adsorption at room temperature under oxidizing conditions (Figure S5d), suggesting a distinct surface environment during reaction. Concurrently, the intensity of carbonate-related bands increases, and additional shoulders at 1455 cm^–1^ and 1390 cm^–1^ become apparent, which are indicative of monodentate carbonate formation. For LFM, the broad band at 1760 cm^–1^ has previously been attributed to bridged carbonate species by Tascon et al. on LaFeO_3_-based perovskites.^32^ Upon increasing the temperature to 400 °C, a further rise in the intensity of gas-phase CO_2_ and a concurrent decrease in N_2_O are observed for both catalysts, consistent with the catalytic performance trends shown in Figure 1. At this temperature, monodentate carbonates become the predominant surface carbonate species. Simultaneously, the intensity of the nitrosyl band on L_0.7_FM diminishes, accompanied by a shift in the peak maximum to 1775 cm^–1^, indicating changes in the local coordination environment. Additionally, a new band emerges at 2178 cm^–1^ for L_0.7_FM (2171 cm^–1^ for LFM). While this region partially overlaps with the absorption of gas-phase CO, the band is clearly distinguishable and is attributed to the formation of surface-bound isocyanate (NCO) species, in line with previous reports.^35,36^

At 500 °C, the intensity of the gas-phase CO_2_ signal continues to increase, while no N_2_O formation is detected, indicating a shift toward complete conversion. The amount of adsorbed NCO species also increases, and monodentate carbonates remain the predominant surface carbonate species on both catalysts. In the case of L_0.7_FM, the nitrosyl band decreases significantly, and a new band appears around 1990 cm^–1^, which can be attributed to molecularly adsorbed CO on more strongly reduced Fe-sites,^37,38^ or adsorbed CO species on CN sites.^39^ In contrast, this CO adsorption feature is absent in the LFM sample, representing a key distinction between the two materials. These observations highlight the critical role of Fe^2+^ surface enrichment in L_0.7_FM. At temperatures below 300 °C, these Fe-sites are readily reduced by CO, enabling the regeneration of Fe^2+^ sites that serve as active centers for nitrosyl adsorption.

Next, we microscopically characterize the L_0.7_FM perovskite after catalysis to draw conclusions about the catalytically active species in the deNO_*x*_ reaction.

### 2.3 Identification of Catalytically Active State

Figure 4a shows an HAADF overview that provides atomic scale insights into the catalyst’s morphology and composition after the NO + CO reaction. The perovskite particles measure several tens of nanometers after catalysis and show signs of additional nanoparticle formation on the surface, as observed in the orange square in Figure 4a. Quantitative, atomically resolved EDXS in Figure 4b–e prove that after catalysis Fe-rich particles are embedded in the perovskite surface. The Fe-rich nanoparticles and A-site-deficient regions are distributed heterogeneously rather than uniformly covering the perovskite surface (see Figure S6). The elemental line profile in Figure 4f, taken along the white arrow in Figure 4e, shows the transition from the stoichiometric bulk to the off-stoichiometric surface of the perovskite (i.e., the A-site deficiency). Note that although atomic fractions provide an intuitive understanding, they can lead to an inaccurate representation of the quantity of the analyzed material. To address this issue, Figure S7 shows the background-corrected net counts of the elemental peaks. Elemental analysis of Figure 4f shows that the Fe-rich regions consist of ∼2 nm large FeO_*x*_ nanoparticles. Minor traces of Mn and La are likely due to residual X-ray signals from the proximity to the Mn- and La-containing perovskite.

**Figure 4:**
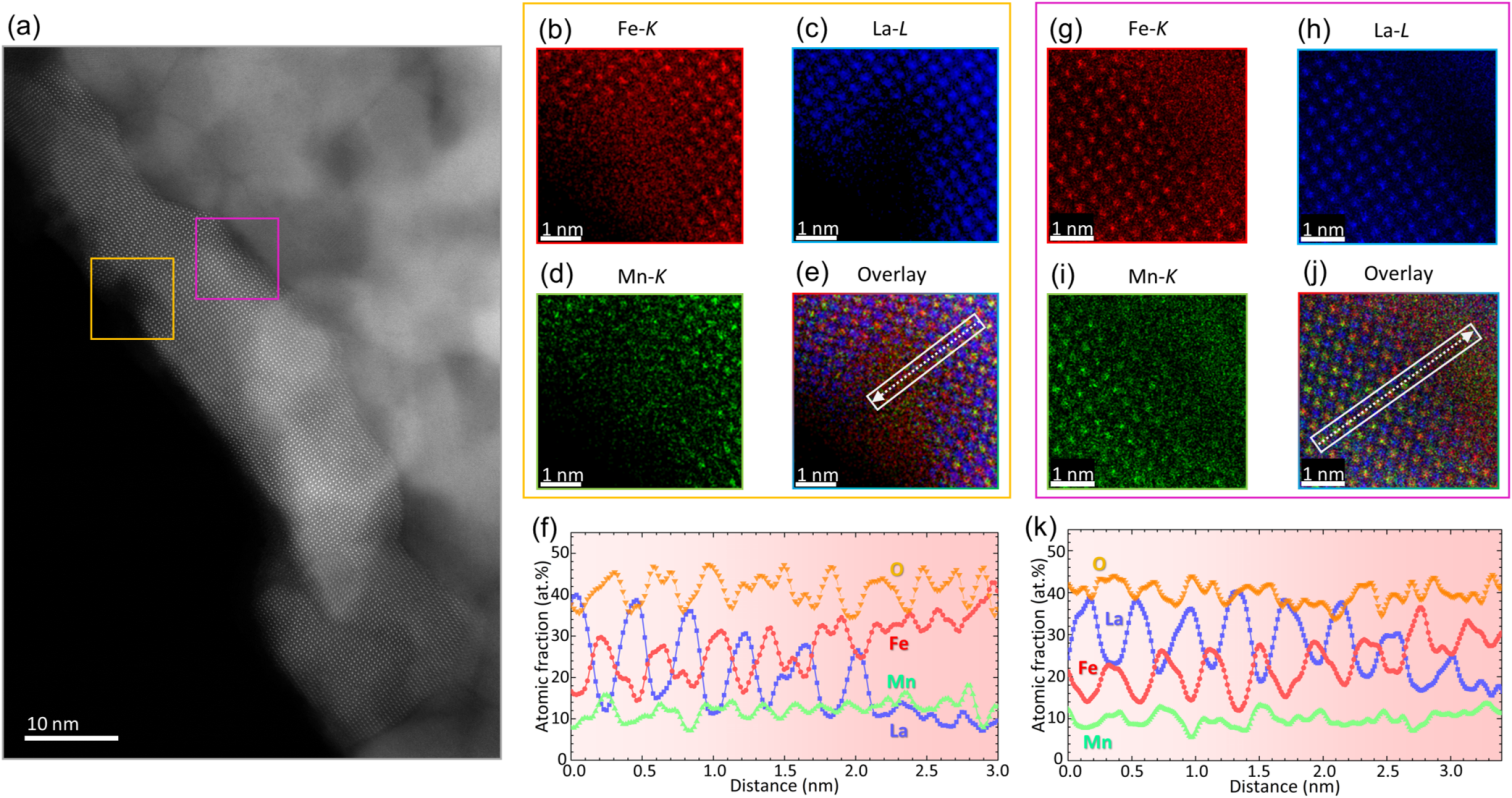
Atomic scale insights into the defects of the L_0.7_FM catalyst after catalysis. (a) HAADF overview image highlighting two squares (orange and pink), where quantitative atomic scale EDXS measurements have been performed. In the orange square, the atomically resolved nanoparticle is visualized along the [100] zone axis. (b, c, d, e) Atomically resolved elemental mappings of Fe, La, Mn, and the overlay of these three mappings, respectively, showing the stoichiometry near the surface of the nanoparticle. (f) Elemental line profile along the white rectangle of panel e highlighting the changes in atomic fraction of O (orange downward triangles), La (blue squares), Fe (red circles), and Mn (green upward triangles). Errors for elements in the perovskite in panel (f) are ± 4 at. % for Fe and Mn, ± 2 at. % for La, and ± 6 at. % for O. Errors for the elements in the FeO_*x*_ particle in panel (f) are ± 9 at. % for Fe, ± 8 at. % for Mn, ± 5 at. % for La, and ± 12 at. % for O. Within the pink frame, (g–j) show atomically resolved elemental mappings of Fe, La, Mn, and the overlay of these three elements, respectively, showing the stoichiometry at the interface to another particle. (k) Elemental line profile along the white rectangle of panel j highlighting the changes in atomic fraction of O, La, Fe, and Mn. Errors for the elements in the perovskite particle in panel (k) are ± 3 at. % for Fe, ± 2 at. % for Mn, ± 3 at. % for La, and ± 4 at. % for O. Errors for the elements in the FeO_*x*_ particle in panel (k) are ± 4 at. % for Fe, ± 2 at. % for Mn, ± 2 at. % for La, and ± 5 at. % for O. Note that the sample thickness was estimated to be 10 nm.

This highlights our third major finding, which is the identification of the catalytically active species: FeO with some undefined amount of oxygen, hence, FeO_*x*_ nanoparticles supported on a strongly A-site deficient La_0.7‑*x*_Fe_0.7_Mn_0.3_O_3_ matrix. These results are in good agreement to the DRIFTS data, where the Fe^2+^ has been interpreted as a possible reactive site for a MvK-type CO reaction. In addition, the elemental analysis in Figure 4f,k shows that the La-poor La_0.7–*x*_Fe_0.7_Mn_0.3_O_3_ is roughly three unit cells thick. The observation of FeO_*x*_ in the spent A-site deficient catalyst strongly suggests that these surface nanoparticles are the catalytically active species, as FeO_*x*_ is known to be an active catalyst for NO reduction,^40,41^ whereas the stoichiometric LFM perovskite does not show such FeO_*x*_ particles, cf. Figures 1 and S7. A similar trend is visible in Figure 4g–k, where the interface of an oriented- and a nonoriented nanoparticle is visualized via atomically resolved EDX measurements. In addition, the catalysts after catalysis were examined by EDX on a larger scale, confirming the presence of A-site deficiency, cf. Figure S8.

Next, we characterize the strain state of the near-surface support material at the FeO_*x*_ interface. We analyze the strain states of the A-site deficient perovskite at grain boundaries and in the final active state FeO_*x*_ particles supported on an A-site deficient perovskite.

The local strain states are analyzed by using geometric phase analysis (GPA) on aberration-corrected HAADF images in L_0.7_FM before and after catalysis.^42^ All analyzed perovskite particles are oriented along the [100] zone axes. To map the lattice distortions, we employed a self-written Python script that applies GPA through a customized cosine-mask filtering approach.^43^ By selectively isolating the relevant diffraction spots for each grain, this script enables strain analyses.

Figure 5a focuses on two adjacent grains along the [100] zone axis in the sample before catalysis, highlighting the pronounced Fe-rich defect formations at the grain boundary. The corresponding fast Fourier transformations (FFTs) in Figure 5b,d, show a shift between the (010), (011), and (001) reflections due to misorientation between the grains. To quantify the strain from our HAADF images, specific diffraction peaks, (010) and (011) reflections, for each grain are highlighted by red circles in Figure 5b, and green circles in Figure 5d. The strain in the *y*-direction of the image (ε_*yy*_ strain component) in Figure 5c,e, expressed here in percentage, shows that the strain is exclusively present at the perovskite grain boundary, which is due to sudden changes of the lattice orientation. These sudden changes (jump from the red to the green spots or vice versa in Figure 5b,d) are presented as artifacts in the GPA, since the method assumes a single smoothly varying lattice. By contrast, the clear absence of strain in the A-site deficient regions is highlighted by dashed red and dashed green areas. We conclude that the system relaxes with the formation of FeO_*x*_ defects instead of a strained A-site deficient perovskite. We identified these FeO_*x*_ nanoparticles as the additional catalytically active species for the A-site deficient L_0.7_FM catalyst.

**Figure 5:**
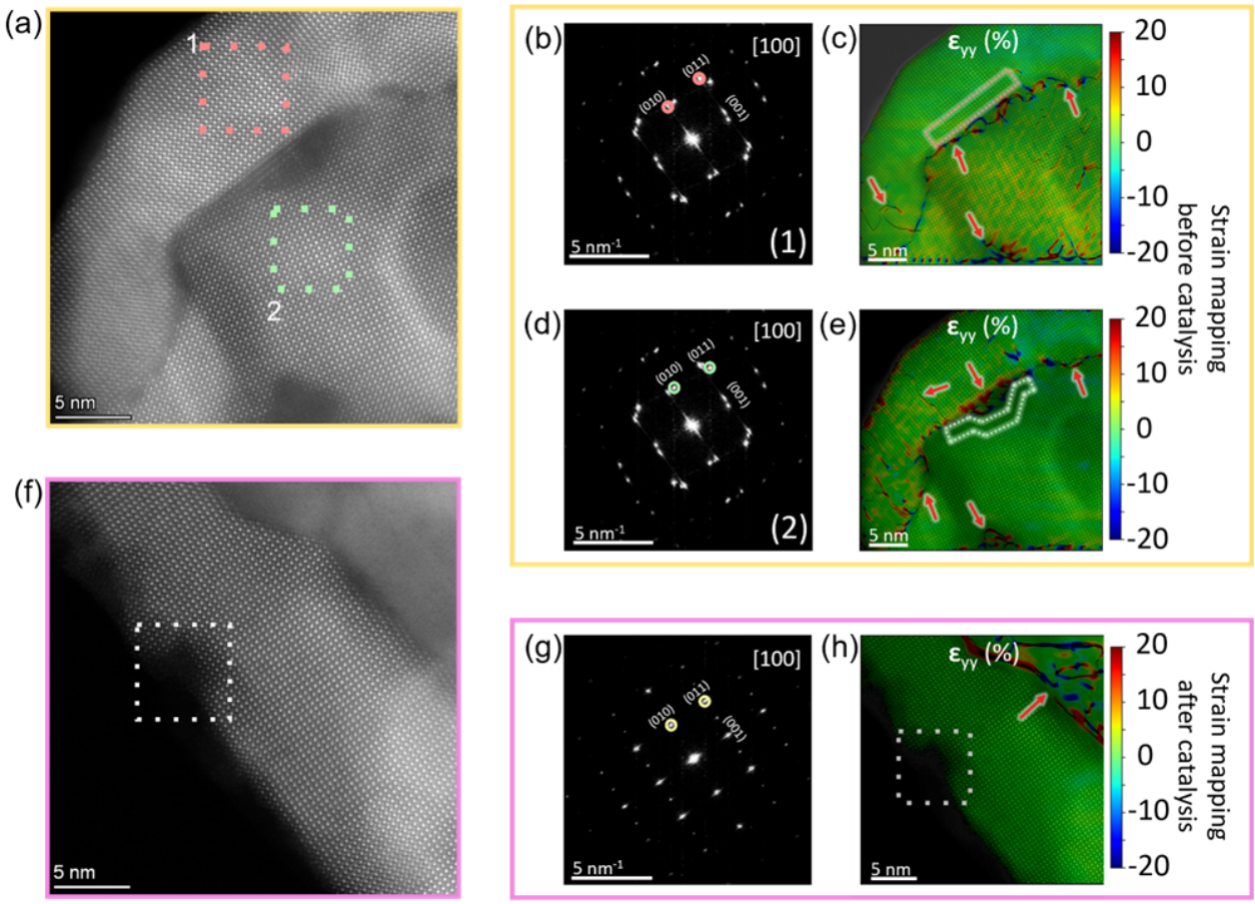
GPA revealing strain-free A-site deficient perovskite before and after catalysis. (a) HAADF–STEM image of L_0.7_FM before catalysis, recorded along the [100] zone axis, highlighting a grain boundary region. (b) FFT of (a), with red circles selecting diffraction spots belonging to the position 1; these spots are used for geometric phase analysis (GPA). (c) Strain map (ε_*yy*_, in percent) calculated from the spots in (b), revealing strain free A-site deficient perovskite. The dashed red box highlights the strain free A-site deficient perovskite. (d) FFT of (a) again, but showing green circles for diffraction peaks of position 2. (e) Strain maps obtained from the spots of position 2, likewise plotted in percent. (f) HAADF–STEM image recorded along the [100] zone axis after catalysis highlighting a FeO_*x*_ nanoparticle at the surface of the perovskite substrate. The dashed white box highlights the strain-free A-site deficient perovskite. (g) FFT of (f), with yellow circles marking reflections chosen for GPA after catalysis. (h) ε_*yy*_ map overlaid with the HAADF image in (f) with the strain in percentage, showing strain-free A-site deficient perovskite. Note that the red arrows point out artifacts of the strain maps, which appear in the nonoriented areas of the sample, such as the area at the grain boundary between the two nanoparticles.

Figure 5f presents an overview HAADF image of the L_0.7_FM catalyst after catalysis. The strain maps in Figure 5h (obtained from circles shown in the FFT in Figure 5g) reveal that the interface of the FeO_*x*_ and the A-site deficient perovskite as well as the interface between the A-site deficient perovskite and the stoichiometric perovskite is strain-free. Noteworthy, the absence of significant strain in the A-site deficient perovskite matches our XRD results (Figure S9), where only slight changes between the two states before and after catalysis are observed. The residual microstrain ε (Figure S9 and Table S2) derived from the peak broadening of the (002) reflection in the diffractograms is more prominent before catalysis and can therefore be attributed to elastic strain due to smaller particle sizes and higher surface stress.

Figure 6a shows that the Fe oxidation state is generally lower in the A-site deficient L_0.7_FM compared to the stoichiometric LFM perovskite, indicating that the iron sites in L_0.7_FM are more easily reduced. For this analysis, a linear calibration curve was obtained using standard samples of FeTiO_3_ and Fe_2_O_3_. This enhanced reducibility can be attributed to the presence of A-site vacancies, which modify the local electronic environment and facilitate the transfer of electrons to the Fe sites. As a result, L_0.7_FM exhibits a higher propensity for reduction under reactive conditions, highlighting the critical role of A-site deficiency in tuning the redox behavior of perovskite materials. The Mn valence in both samples was evaluated using a calibration curve obtained from standard samples of MnO, Mn_2_O_3_, and MnO_2_, fitted with a quadratic function (dashed black line) shown in Figure 6b. In the A-site deficient L_0.7_FM perovskite, no significant change in the Mn oxidation state is observed, suggesting that the Mn sites remain largely unaffected during catalysis. In contrast, the Mn valence in the stoichiometric LFM sample increases after catalytic treatment, indicating partial oxidation of Mn. This difference implies that L_0.7_FM may form a higher concentration of oxygen vacancies during reduction or reaction than LFM as confirmed by temperature-programmed hydrogen reduction or in situ XRD.^16^ In due course, the enhanced oxygen mobility associated with A-site deficiency facilitates reduction at Fe sites while maintaining the Mn valence relatively stable. To emphasize the surface-bound reduction propensity of L_0.7_FM, Figure 6c presents the Fe 2p region during in situ XPS analysis of the L_0.7_FM sample under 0.3 mbar CO at 100 °C, 500 °C, and after cooling to 25 °C. At 100 °C, a peak at 710.6 eV indicates that the initial oxidation state of iron in the CO atmosphere is close to Fe^3+^. Upon heating to 500 °C, a new peak emerges at 706.8 eV, corresponding to metallic iron formation. Cooling the sample back to 25 °C in CO leads to the disappearance of the metallic iron peak, indicating surface reoxidation. Since no gas-phase oxygen is present, this observation suggests that oxygen is supplied from the perovskite bulk to the surface iron species, thereby indicating the formation of oxygen vacancies in the perovskite. Note that the corresponding raw spectra are presented in Figure S10.

**Figure 6:**
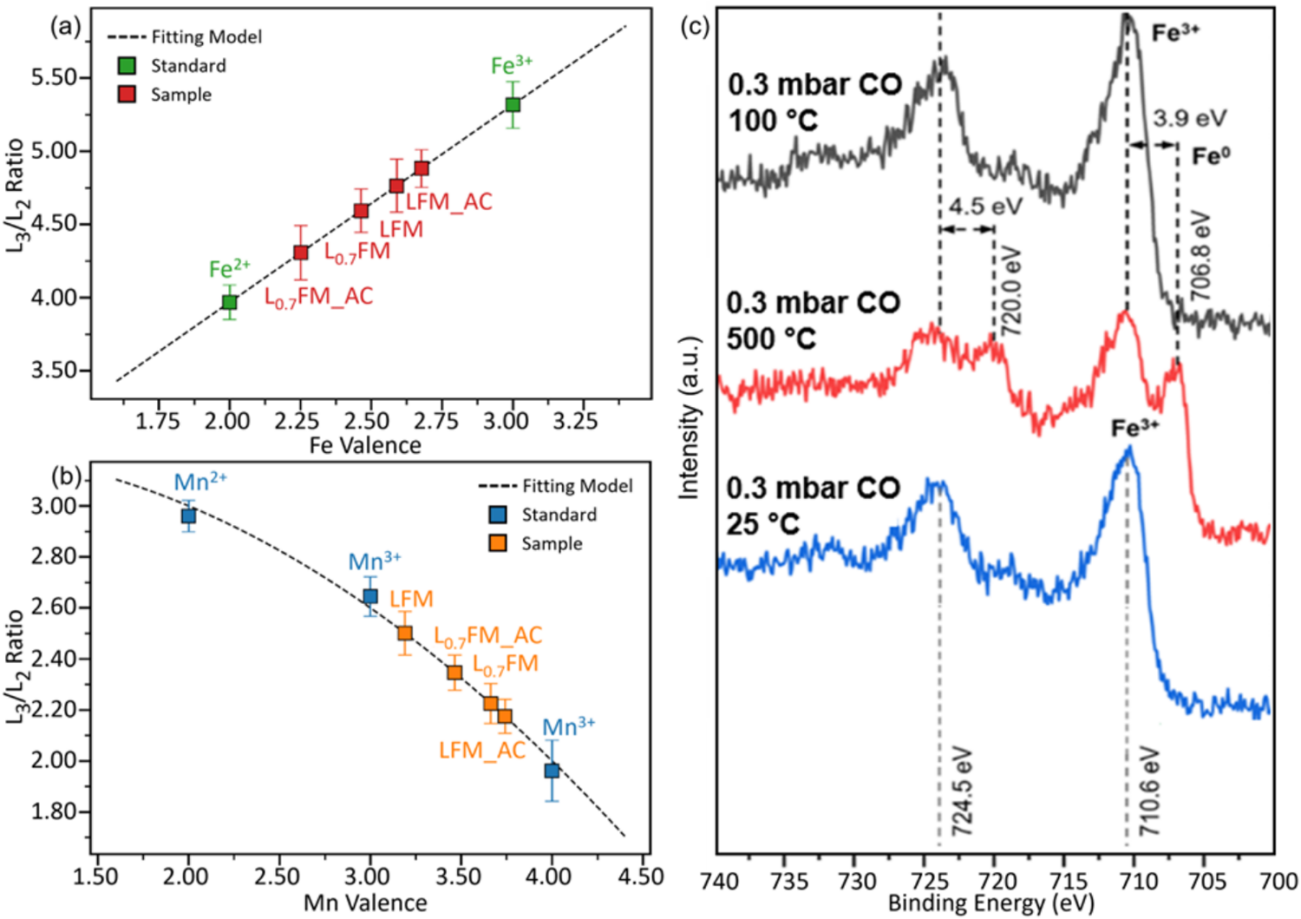
Electronic states of perovskites before, during and after catalysis. (a) Valence quantification of Fe using ELNES. The two standards (shown in green) FeTiO_3_ and Fe_2_O_3_ are used to obtain reference values for Fe^2+^ and Fe^3+^, respectively. (“AC” designates after catalysis.) (b) Valence quantification of Mn using ELNES. The three standards (blue) MnO, _Mn2O3_ and MnO_2_ are used to obtain reference values for Mn^2+^, Mn^3+^, and Mn^4+^, respectively. (c) In situ and ex situ XPS measurements obtained at different temperatures reveal a pronounced change in the Fe oxidation state. This variation is evidenced by significant shifts in the Fe 2p_3/2_ and Fe 2p_1/2_ peaks when comparing low temperatures (25 and 100 °C) with high temperature (500 °C) under a CO atmosphere.

## 3 Discussion

Our findings suggest that the off-stoichiometry in our model system of A-site deficient perovskites originates from heterogeneously distributed A-site deficiencies at surfaces and particle interfaces, while the bulk of the particles remains stoichiometric with no detectable A-site deficiency, cf. Figure 7. The formation of FeO_*x*_ occurs both at A-site–deficient grain boundaries and at A-site–deficient surfaces in the form of individual nanoparticles.

**Figure 7:**
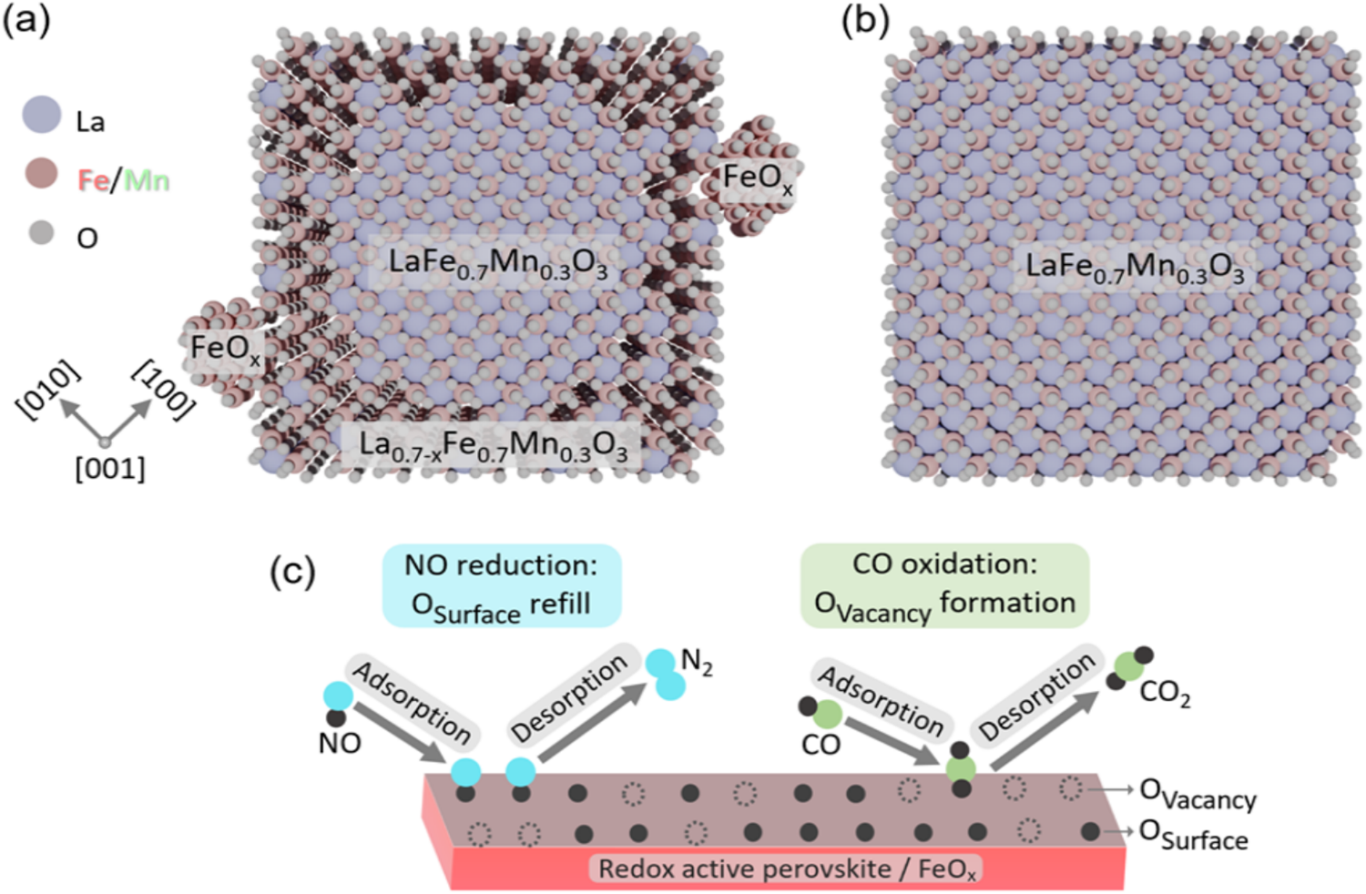
Proposed model structure for the A-site deficient perovskite L_0.7_FM in comparison to LFM. (a) L_0.7_FM consisting of stoichiometric bulk LFM transitioning to A-site deficient La_0.7‑*x*_Fe_0.7_Mn_0.3_O_3_ areas at the surface, where catalytically active FeO_*x*_ nanoparticles are embedded. (b) Stoichiometric LFM for comparison. Blue, red and gray balls represent La, Fe, and oxygen atoms, respectively. (c) Schematic representation of the proposed MvK mechanism on the redox active sites of the perovskite and the FeO_*x*_ particles.

Furthermore, the correlative microscopic and gas reaction analysis indicate that these FeO_*x*_ nanoparticles embedded in strain-free A-site deficient La_0.7–*x*_Fe_0.7_Mn_0.3_O_3_ are responsible for the earlier onset temperatures in the catalytic activity in comparison to the non-A-site deficient catalyst. We identify Fe^2+^ species as the catalytically active site in a MvK-type reaction pathway of the CO oxidation. In our proposed catalytic mechanism concluded from correlated in situ DRIFTS, in situ XPS, EDX and EELS measurements, CO oxidation to CO_2_ and NO reduction to N_2_ happens at lower temperatures on the FeO_*x*_ compared to the Fe-sites inside the perovskite. Note that both active sites are catalytically active in the A-site deficient catalyst, whereas only the Fe-site of the perovskite is active in the stoichiometric catalyst as shown in Figure 7c. Additionally, at high temperatures, the adsorption of CO on more reduced Fe sites plays a decisive role in promoting catalytic activity. The uniform distribution of Mn throughout the perovskite suggests that Mn primarily contributes to lattice stabilization when substituted for Fe. The stable Mn valence in L_0.7_FM, combined with the reduced Fe valence and the reoxidation of metallic Fe in an O_2_-free environment, indicates the formation of oxygen vacancies in the A-site-deficient perovskite. This conclusion is supported by the observation that the FeO_*x*_ nanoparticles observed at the surface are composed mainly of Fe and oxygen, with only residual amounts of Mn and La. Our strain results are in good agreement with the existing XRD results, where no lattice distortion could be observed even for higher concentrations of La-deficiencies. Based on our quantitative chemical, electronic and strain states before and after catalysis, we conclude that the La- deficient perovskite is only a few unit cells thick and remains stable down to ∼50 at. % of La-deficiencies. Additionally, no observable changes in lattice parameters are measured in the A-site deficient phase and the system relaxes via the formation of FeO_*x*_ nanoparticles. We further conclude that the indistinguishable XRD patterns of La-deficient perovskites and their stoichiometric counterparts stem from the fact that La vacancies introduce no appreciable long-range lattice distortion. Likewise, the spatially heterogeneous distribution of rather small FeO_*x*_ nanoparticles prevents their detection by standard XRD methods. The crystallinity of the FeO_*x*_ nanoparticles remains an open question, since diffraction measurements using STEM and XRD failed to detect crystalline phases. However, the presence of partial structural ordering cannot be excluded, and the nanoparticles may exhibit short-range order despite the absence of long-range crystallinity.

Our findings on La_0.7_Fe_0.7_Mn_0.3_O_3_ establish a structural and mechanistic foundation for future studies of A-site deficient perovskite catalysts. In particular, our findings highlight the need for atomic scale characterization when conventional techniques, such as XRD, reveal only subtle or negligible differences compared to stoichiometric analogues. The structure–property relationships uncovered in this study are expected to be broadly relevant to A-site deficient oxides and may provide a valuable framework for understanding the role of defects, such as A-site vacancies, in governing catalytic activity.

## Supplementary Material



## Notes

Experimental details; TEM images and quantitative EDXS mappings; catalytic conversion and product formation data; DRIFT spectra; quantitative ICP and XPS results, raw EELS spectra, powder XRD data including strain analyses; and table with quantitative XRD results (PDF)

## Abbreviations

deNO*x*: denitrification of NOx
L_0.7_FM: La_0.7_Fe_0.7_Mn_0.3_O_3_
LFM: LaFe_0.7_Mn_0.3_O_3_
BC: before catalysis
AC: after catalysis
XRD: X-ray diffraction
STEM: scanning transmission electron microscopy
HAADF: high-angle annular dark-field
EDXS: energy-dispersive X-ray spectroscopy
DRIFTS: diffuse reflectance infrared Fourier transform spectroscopy
GPA: geometric phase analysis
FFT: fast Fourier transformation
MvK: Mars-van Krevelen
EELS: electron energy-loss spectroscopy
ELNES: energy-loss near-edge spectroscopy
ICP: inductively coupled plasma
XPS: X-ray photoelectron spectroscopy
