# Supplementary material for: Defect-Engineered Perovskites: Atomic Scale Nature of A‑Site Vacancy-Stabilized Catalytically Active Phase
Source: J Am Chem Soc. 2026 Mar 2;148(9):9980–9. doi: 10.1021/jacs.5c22657 (PMC12983319; doi:10.1021/jacs.5c22657)
Supplement: Supplementary file 1 [file ja5c22657_si_001.pdf]

SI: Defect-Engineered Perovskites: Atomic Scale Nature of A-Site Vacancy-Stabilized Catalytically Active Phase

Roham Talei[1†], *Asghar Mohammadi*[2†], *Thomas F. Winterstein*[2], *Christoph Malleier*[2], *Guido Schmitz*[1], *Simon Penner*[2], *Nicolas Bonmassar*[*,1]

[1] Department of Materials Physics, Institute for Materials Science, University of Stuttgart, Heisenbergstr. 3, 70569 Stuttgart, Germany

[2] Department of Physical Chemistry, University of Innsbruck, Innrain 52c, A-6020 Innsbruck, Austria

[†] These authors contributed equally.

[*] To whom correspondence should be addressed: Nicolas.Bonmassar@imw.uni-stuttgart.de

1.0 Methods

Sample Synthesis

Two lanthanum iron manganite perovskite samples were synthesized employing a sol-gel approach. The concentrations of the metal nitrate precursors ($La(NO_3)_3{\cdot}6H_2O$, $Fe(NO_3)_3{\cdot}9H_2O$, and $Mn(NO_3)_2{\cdot}4H_2O$) were chosen to achieve nominal compositions of stoichiometric $LaFe_{0.7}Mn_{0.3}O_3$ (LFM) and A-site deficient $La_{0.7}Fe_{0.7}Mn_{0.3}O_3$ ($L_{0.7}FM$). Glycine was added in a 1:1 ratio of Glycine: NO_3^- as a complexing agent. The resulting aqueous solution was heated for approximately three hours at 90 °C, turning it into a viscous gel. Further heating to 240 °C resulted in the formation of metal oxides and carbon residues under a controlled exothermal reaction. Calcination at 700 °C for 5 hours in air removed the remaining carbon, simultaneously forming the perovskite structure, as evidenced by XRD.

X-Ray Diffraction and Rietveld Refinement

X-ray diffraction (XRD) patterns of the samples were recorded using a BRUKER D8 DISCOVER diffractometer with a LynxEye XE 1D silicon strip detector and Cu-K_α radiation. Powder samples were fixed on glass sample holders and measured from 5 - 90° 2θ with a 0.01° step width, while continuously rotating the sample. Rietveld refinement was carried out using the TOPAS 7.0 program with a full axial model. A Double-Voigt approach and a combination Lorentz & Gauss models were used within TOPAS to calculate crystallite size and micro strain concurrently. The resolution function of the diffractometer was obtained from structural refinement of a NIST LaB6 standard.

Scanning Transmission Electron microscopy

Specimen preparation was performed by a simple drop-casting technique, wherein a suspension of perovskite particles in isopropanol was deposited onto lacey carbon grids. High-resolution scanning transmission electron microscopy (STEM) analyses were conducted using a probe-corrected Thermo Fisher Scientific Spectra 300 S/TEM, with sub-angstrom imaging capabilities and advanced analytical features, such as high-angle annular dark field (HAADF) imaging, was employed alongside energy-dispersive X-ray spectroscopy (EDX) using the Super-X EDS system with improved signal-to-noise ratio for oxygen quantification. To reduce radiolysis in our transition metal oxide samples, we used the

instrument at 300 kV for all measurements. This configuration offers specimen-tilt independence and high sensitivity, facilitating accurate elemental mapping. Quantitative EDX analysis was performed utilizing the Schreiber–Wims ionization cross-section, optimized spectrum fit, absorption correction to minimize matrix effects, estimated sample thicknesses (10-50 nm depending on sample area and verified by electron energy-loss spectroscopy (EELS) t/ λ measurements) and a parabolic background model, all integrated into the Thermo Scientific Velox software. The errors for the different areas in the elemental line scan (Figure 2 and Figure 4) were determined by using the spectrum integration function with an integration width of one pixel and the same lengths and the same parameters as we used for the atomic fraction calculation. The strain in Figure 4 was quantified by a self-written python script, which is explained in more detail elsewhere. [1]

EELS data were obtained in diffraction mode using a EELS Continuum S detector at 300 kV, 22 mrad convergence semi-angle, and a 0.15 eV/channel dispersion leading to an overall energy resolution of 1.5 eV. Valence quantifications obtained from the Fe-L_3/L_2 and Mn-L_3/L_2 ratios were carried out using a double-arctangent background subtraction. For this procedure, the height of the L_3 edge bacvkground was set to two-thirds of the total L_3+L_2 background height, while the height of the L_2 edge background was set to one-third of the L_3+L_2 background. The white line ratios were calculated by energy windows with a width of 5 eV. By shifting these windows within our energy resolution of 1.5 eV, we determined the standard deviations for our L_3/L_2 ratios. Standard measurements of $FeTiO_3$, Fe_2O_3, MnO, Mn_2O_3 and MnO_2 were performed to calibrate the Fe^{2+}, Fe^{3+}, Mn^{2+}, Mn^{3+}, and Mn^{4+} oxidation states, respectively.

Diffuse reflectance Fourier-Transform infrared spectroscopic (DRIFT) measurements of NO and CO single and co-adsorption

The NO and CO adsorption experiments were conducted on powder samples placed in a heated DRIFT cell (PIKE) equipped with CaF_2 window and mounted on a Agilent Cary 660 FT-IR spectrometer. The as-prepared catalysts were placed in an alumina sample holder (Diffuse IR HC Porous Ceramic cup, 6.0 mm OD, 4.0 mm height, 4.7 mm ID and 2.0 mm depth) and pretreated at 500 °C in He or O_2 (for oxidative pre-treatment) with a total flow rate of 80 mL min^{-1}. A background spectrum (60 scans) was recorded at each desired temperature mainly under He flow during cooling the sample after the pre-

treatment phase. NO and CO (both 5% in He) adsorption was carried out at 25 °C or selected elevated temperatures with the constant total flowrate of 80 ml min^{-1}. Kubelka-Munk normalized spectra (30 scans) were collected as a function of adsorption time or temperature to monitor the evolution of adsorbed species during the NO/CO single or co-adsorption. For the spectra during heating, corresponding background spectra recorded at the same temperature and He flow were used.

Catalytic activity in the reduction of NO by CO

200 mg of catalyst powder was fixed with quartz wool in a home-made 10 mm (inner diameter) quartz fixed-bed flow reactor setup under atmospheric pressure and a total flow rate of 200 mL min^{-1} (CO: NO: He =1:1:98 mL min^{-1}, GHSV = 9000 h^{-1}). In each catalytic test, the reactor was heated in a Linn High Therm tube furnace at 2 °C min^{-1} to 500 °C followed by an isothermal period at maximum temperature for 10 min. The output gas was directly detected by infrared spectroscopy of the gas phase (Agilent Cary 660 FT-IR). As the outlet line is already connected to a quadrupole mass spectrometer (Balzers QME 125), the NO conversion results are also double-checked with QMS. N_2 formation rates were determined by difference from the measured rates of NO conversion and of N_2O. For quantification of N_2O external calibration of IR signals has been done using a known composition of N_2O in He. To display the catalytic activity as a function of temperature, we use the following equation to calculate the NO conversion:

$$NO_{Conversion} = 100 * (1 - \frac{[NO]_{out}}{[NO]_{in}})$$

$[NO]_{out}$ and $[NO]_{in}$ indicate the inlet and outlet concentration of NO, respectively. The impact of mass transport limitations in the chosen reactor setup has been thoroughly assessed and found to be negligible. For details of the calculation, we refer to our previous work. [1]

X-ray photoelectron spectroscopy

To investigate the surface electronic structure in situ, NAP-XPS measurements were performed in a customized commercial UHV system (SPECS GmbH). The setup includes a μFOCUS 600 NAP monochromatic small-spot Al Kα X-ray source (100 × 300 μm²), a vertically mounted hemispherical energy analyzer (PHOBIOS 150 NAP), and a μ-metal–shielded analysis chamber to minimize external magnetic field interference. The differentially pumped analyzer enables backfilling of the chamber up

to 30 mbar with various gases or gas mixtures (e.g., NO + CO) supplied via mass-flow controllers (Bronkhorst). For measurements, powdered samples were pressed into pellets placed on a stainless-steel grid and mounted to the sample holder using a front plate for stabilization. An IR laser (IPG Photonics, max. 100 W) positioned beneath the analysis chamber heated the samples from the backside through an 8-mm opening in the holder. Temperature was monitored using a K-type thermocouple attached to the stainless-steel grid inside the pellet. All experiments were conducted in a 1:1 NO/CO atmosphere, with CO (Messer, 4.7 purity) and NO (Linde, 2.5 purity).

Photoelectrons were collected through a 300-µm nozzle positioned in front of the sample, aligned with an 8-mm aperture in the front plate. At mbar pressures, the X-ray–ionized gas region between the sample and nozzle provided charge compensation, eliminating core-level shifts even for poorly conducting samples. The X-ray source was operated at 70 W and 13 kV, and all spectra were acquired under identical conditions, particularly with respect to the pass energy (50 eV). Charging effects were corrected by calibrating all binding energies to the C–C component of the adventitious carbon C 1s peak at 284.8 eV. Data analysis was performed using CasaXPS, applying relative sensitivity factors (RSFs) and electron mean free path corrections for quantification. The following RSFs were used: La 3d: 47.6, Mn 2p: 13.9, Fe 2p: 16.4, and O 1s: 2.93. A pass energy of 50 eV was employed for all measurements.

2.0 SI Figures

SI Table 1: Nominal catalyst composition derived from EDX, *ex situ* XPS, and ICP analysis from Reference [2]. Catalysts after catalysis are marked with AC.

Sample	EDX	XPS	ICP
LFM	$La_{1.00\pm0.09}Fe_{0.66\pm0.08}Mn_{0.26\pm0.04}O_{3.08\pm0.09}$	$La_{1.55}Fe_{0.22}Mn_{0.13}O_{3-\delta}$	$La_{1.07}Fe_{0.67}Mn_{0.26}O_{3-\delta}$
$L_{0.7}$FM	$La_{0.83\pm0.08}Fe_{0.78\pm0.09}Mn_{0.32\pm0.05}O_{3.07\pm0.09}$	$La_{0.90}Fe_{0.43}Mn_{0.18}O_{3-\delta}$	$La_{0.78}Fe_{0.66}Mn_{0.26}O_{3-\delta}$
LFM-AC	$La_{1.02\pm0.09}Fe_{0.67\pm0.08}Mn_{0.28\pm0.04}O_{3.02\pm0.09}$	-	-
$L_{0.7}$FM-AC	$La_{0.84\pm0.08}Fe_{0.79\pm0.09}Mn_{0.33\pm0.05}O_{3.05\pm0.09}$	-	-

Table S2: Unit cell parameters, crystallite size and micro strain as refined from the XRD measurements. There is an uncertainty for all unit cell parameters of ± 0.001 Å and ± 0.1 nm for the crystallite size.

Sample	a [Å]	b [Å]	c [Å]	Crystal Size [nm]	Strain ε
LFM a.c.	5.554	7.807	5.516	28.8	0.404 ± 0.007
LFM 1st cycle	5.558	7.811	5.523	30.4	0.390 ± 0.006
LFM 2nd cycle	5.559	7.811	5.523	30.4	0.382 ± 0.006
$L_{0.7}$FM a.c.	5.553	7.793	5.504	22.8	0.416 ± 0.009
$L_{0.7}$FM 1st cycle	5.553	7.803	5.510	23.8	0.400 ± 0.008
$L_{0.7}$FM 2nd cycle	5.556	7.803	5.512	24.1	0.397 ± 0.008
$L_{0.7}$FM 650 °C	5.544	7.842	5.544	30.1	0.354 ± 0.005

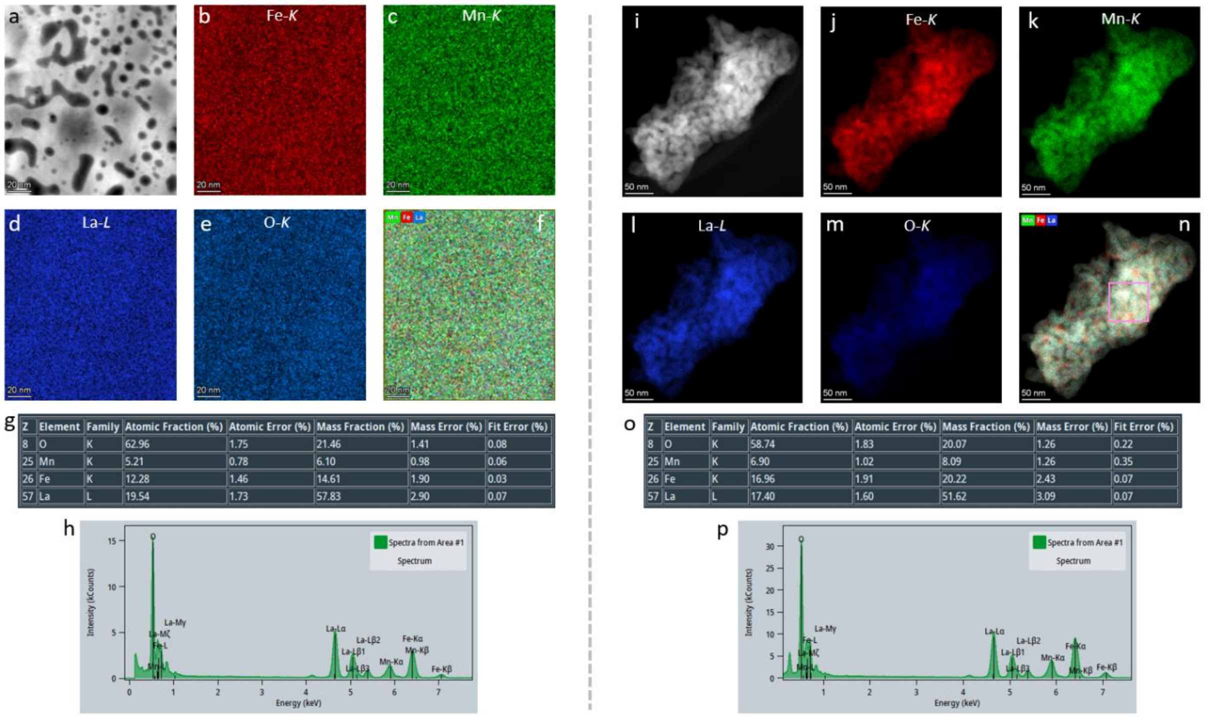

SI Figure 1: Comparison between non-A-site deficient LFM (left) and A-site deficient $L_{0.7}$FM (right). (a) Overview HAADF image of the LFM catalyst. (b) – (e) Elemental mappings of Fe-*K* (red), Mn-*K* (green), O-*K* (dark blue), La-*L* (blue), respectively. (f) Overlay of Mn, Fe, and La signals showing a homogenous distribution. (g) Quantification of atomic fraction and mass fraction of O, Mn, Fe, and La. (h) Corresponding EDX spectrum taken from the whole area of panel a. (i) Overview HAADF image of the $L_{0.7}$FM catalyst. (j) – (m) Elemental mappings of Fe-*K* (red), Mn-*K* (green), O-*K* (dark blue), La-*L* (blue), respectively. (n) Overlay of Mn, Fe, and La signals showing a heterogenous distribution for Fe, indicated by red spots at the interfaces between the nanoparticles. (o) Quantification of atomic fraction and mass fraction of O, Mn, Fe, and La determined from the pink square in panel n. (p) Corresponding EDX spectrum taken from the pink square in panel n. Sample thickness was estimated to be 50 nm in both cases.

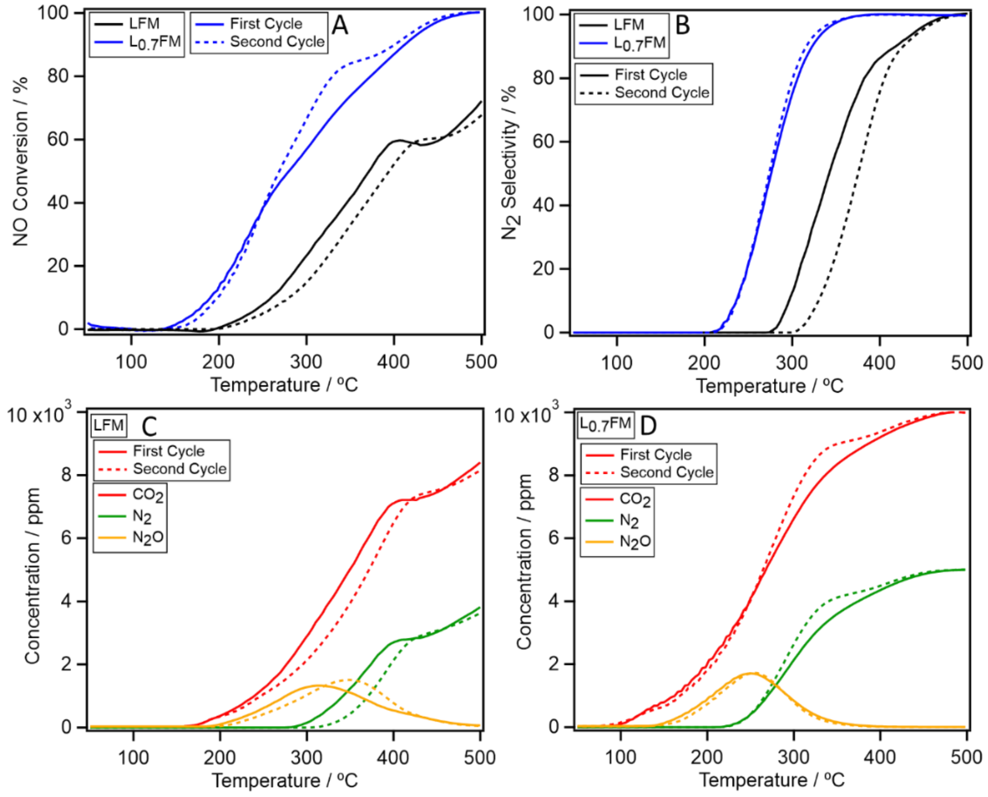

SI Figure 2: NO conversion (Panel A) and N_2 selectivity (Panel B) profiles during first and second catalytic NO reduction by CO cycles on LFM and $L_{0.7}$FM catalysts Product distribution for LFM (Panel C) and $L_{0.7}$FM (Panel D) samples during both reaction cycles. Total gas flow rate: 200 mL min^{-1} with a composition of (CO:NO:He = 1:1:98) of the inlet flow. Heating ramp: 2 °C min^{-1} between 50 °C and 500 °C. Sample mass: 200 mg.

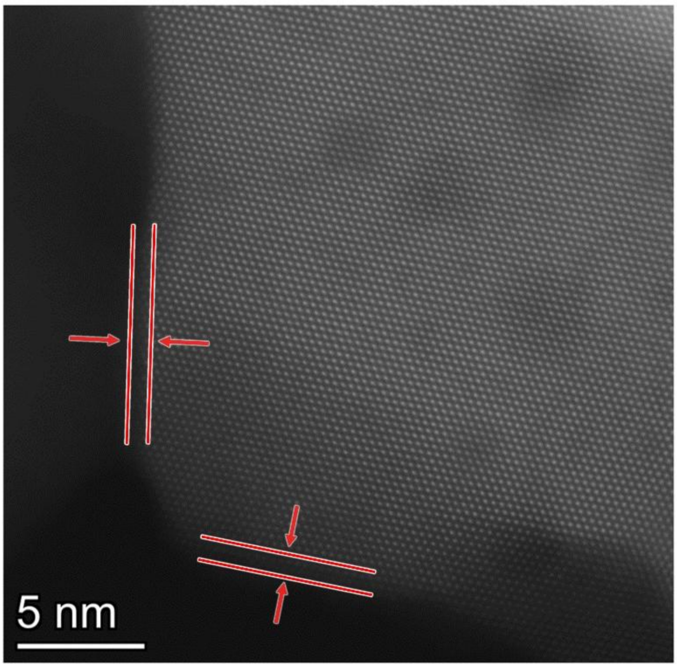

SI Figure 3: ADF image of the stoichiometric LFM. The surface shows an amorphous layer similar to the $L_{0.7}$FM catalyst. The red markings indicate an amorphous surface layer on the catalysts, likely arising from slight surface degradation caused by environmental exposure.

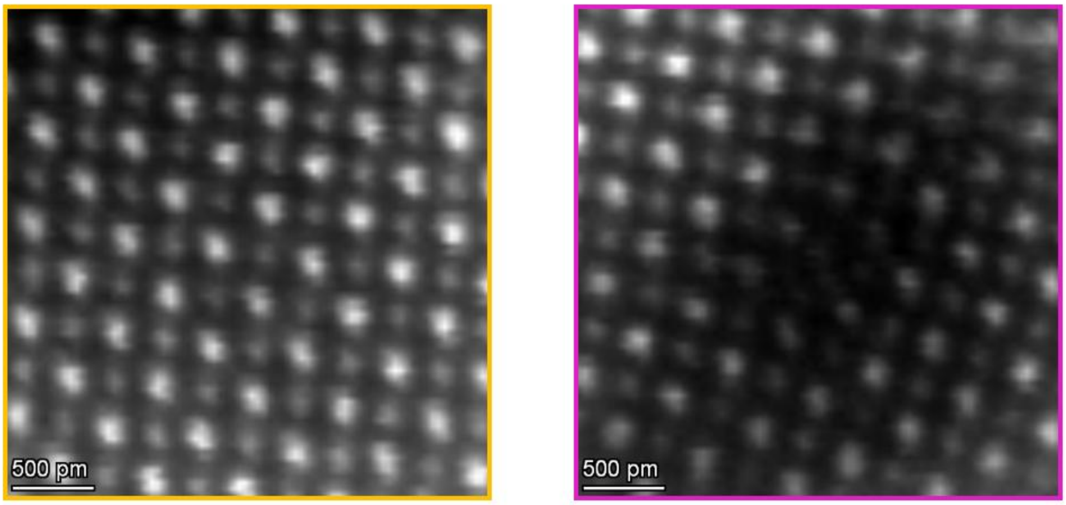

SI Figure 4: ADF images of the orange and pink areas in Fig. 2a after the respective EDX measurements.

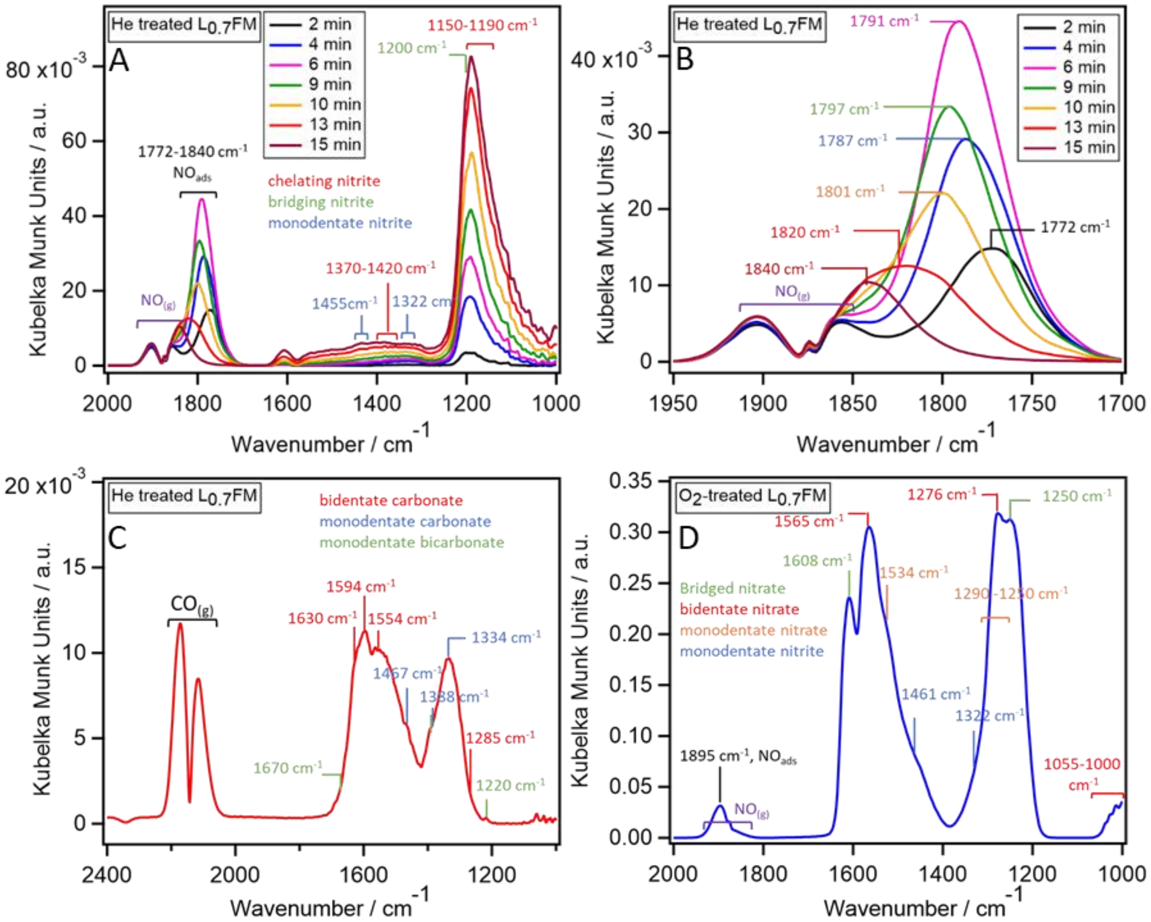

SI Figure 5: (**a**) Time evolution of IR bands during NO adsorption on He-treated $L_{0.7}FM$ at 25 °C. (**b**) Zoom in of the bands at 1700-1800 cm^{-1} region in (**a**). (**c**) DRIFT spectrum of CO adsorption on He-treated $L_{0.7}FM$ at 25 °C after 15 min and (**d**) is the DRIFT spectrum of NO adsorption on O_2-treated $L_{0.7}FM$ at 25 °C after 15 min. The total adsorption flow rate was 80 ml min^{-1} (5% NO or CO in He).

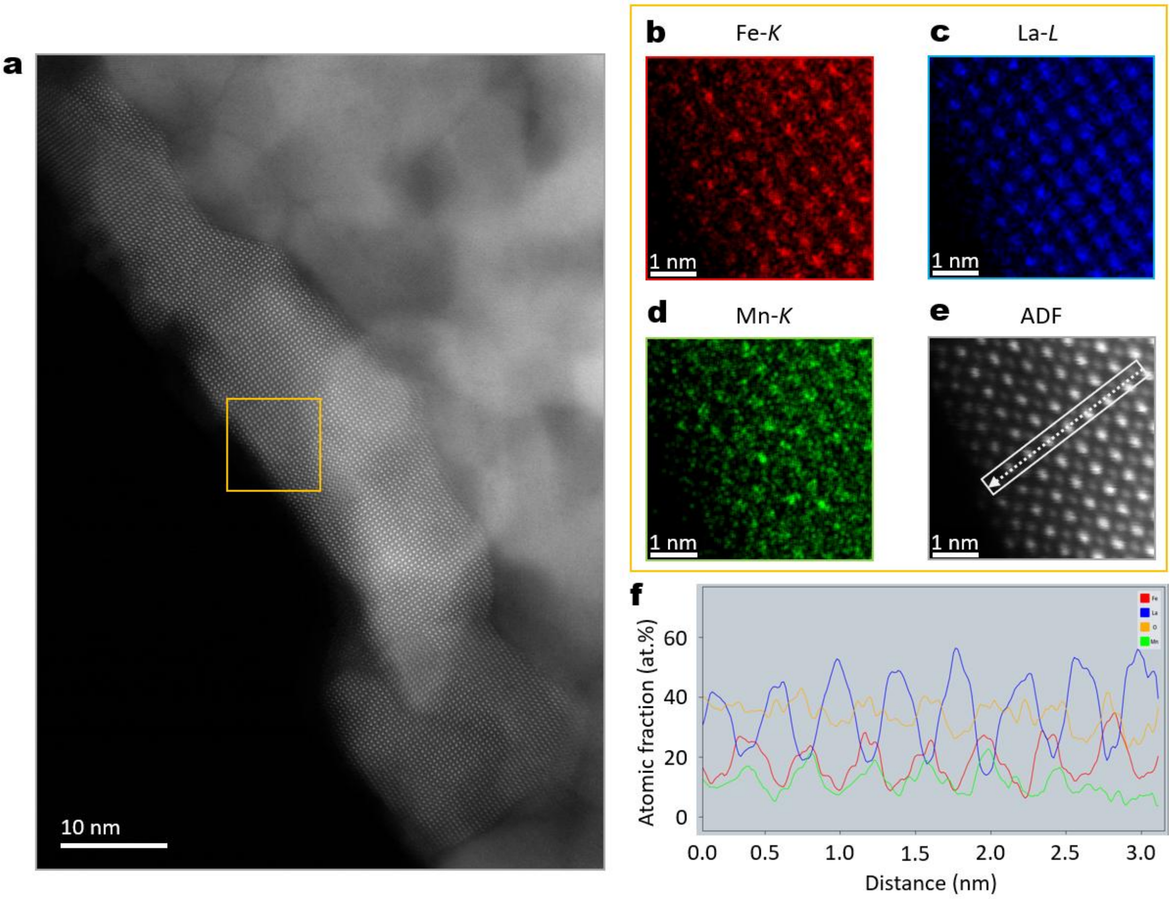

SI Figure 6: (**a**) HAADF overview image highlighting one orange square, where quantitative atomic scale EDXS measurements have been performed. (**b**), (**c**), (**d**), (**e**) Atomically resolved elemental mappings of Fe, La, Mn, and the HAADF image, respectively, showing the stoichiometry near the surface of the perovskite particle. (**f**) Elemental line profile along the white rectangle of panel **e** highlighting the stable stoichiometry in atomic fraction of O (orange), La (blue), Fe (red), and Mn (green).

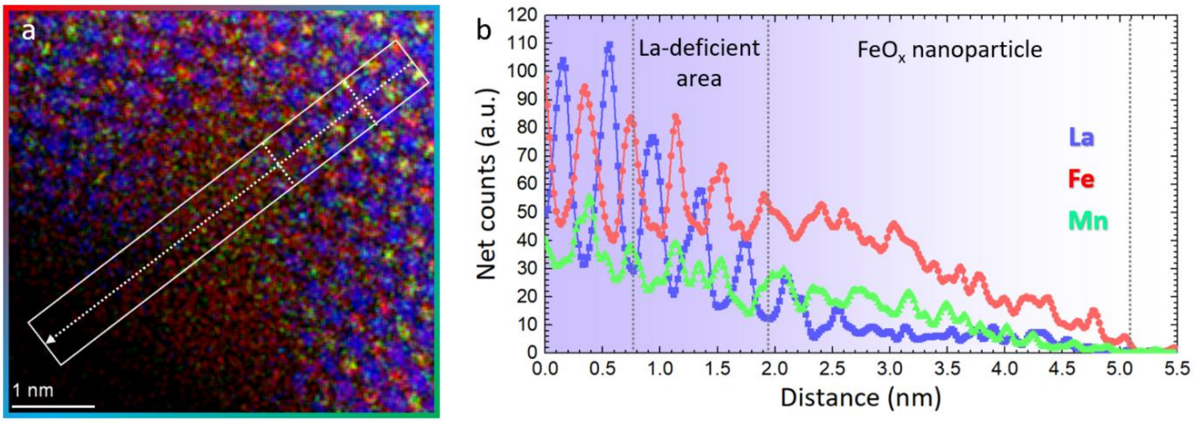

SI Figure 7: (**a**) EDX chemical mapping of the La (blue), Fe (red), Mn (green) distribution. (**b**) Background corrected net intensity of the elemental peaks (La-*L*, Mn-*K*, Fe-*K*) as a function of distance. Panel (**b**) indicates two different interfaces: Interface 1: the stoichiometric perovskite with non-stoichiometric perovskite, Interface 2: the non-stoichiometric perovskite with the FeO_x nanoparticles

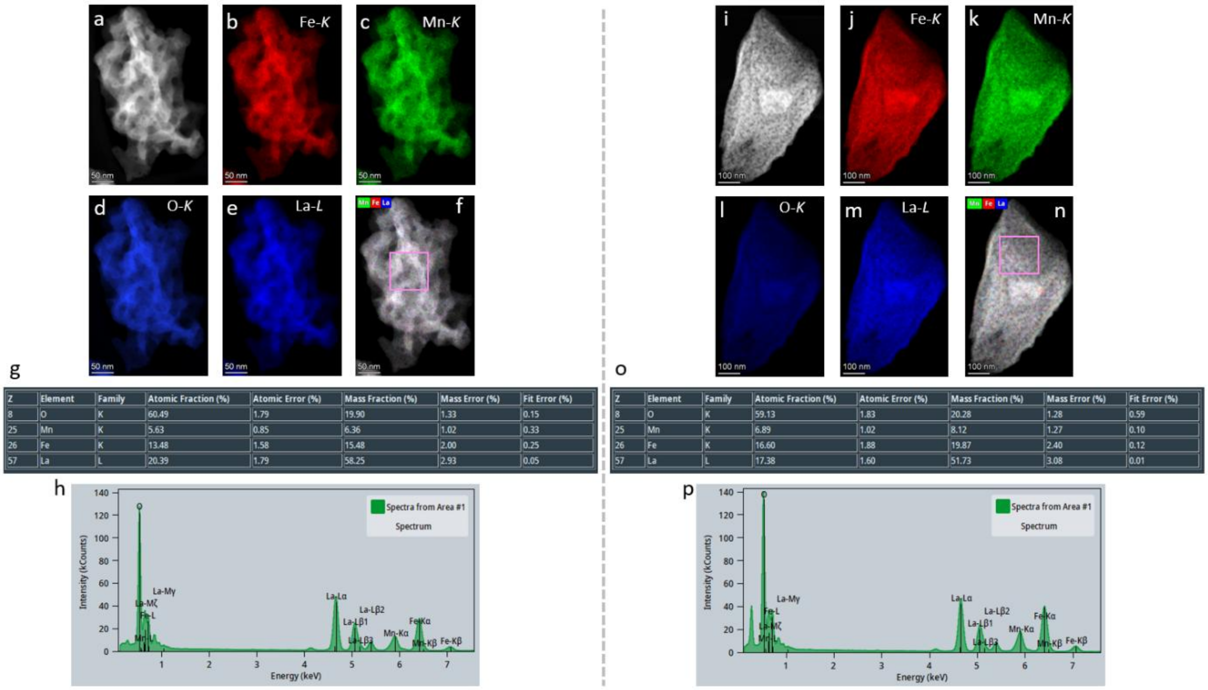

Z	Element	Family	Atomic Fraction (%)	Atomic Error (%)	Mass Fraction (%)	Mass Error (%)	Fit Error (%)
8	O	K	60.49	1.79	19.90	1.33	0.15
25	Mn	K	5.63	0.85	6.36	1.02	0.33
26	Fe	K	13.48	1.58	15.48	2.00	0.25
57	La	L	20.39	1.79	58.25	2.93	0.05

Z	Element	Family	Atomic Fraction (%)	Atomic Error (%)	Mass Fraction (%)	Mass Error (%)	Fit Error (%)
8	O	K	59.13	1.83	20.28	1.28	0.59
25	Mn	K	6.89	1.02	8.12	1.27	0.10
26	Fe	K	16.60	1.88	19.87	2.40	0.12
57	La	L	17.38	1.60	51.73	3.08	0.01

SI Figure 8: Comparison between non-A-site deficient LFM (left) and A-site deficient $L_{0.7}FM$ (right) after catalysis. (a) Overview HAADF image of the LFM catalyst after reaction. (b) – (e) Elemental mappings of Fe-*K* (red), Mn-*K* (green), O-*K* (dark blue), La-*L* (blue), respectively. (f) Overlay of Mn, Fe, and La signals showing a homogenous distribution. (g) Quantification of atomic fraction and mass fraction of O, Mn, Fe, and La. (h) Corresponding EDX spectrum taken from the whole area of panel a. (i) Overview HAADF image of the $L_{0.7}FM$ catalyst. (j) – (m) Elemental mappings of Fe-*K* (red), Mn-*K* (green), O-*K* (dark blue), La-*L* (blue), respectively. (n) Overlay of Mn, Fe, and La signals showing a heterogenous distribution for Fe, indicated by red spots at the interfaces between nanoparticles. (o) Quantitative measurement of atomic fraction and mass fraction of O, Mn, Fe, and La determined from the pink square in panel n. (p) Corresponding EDX spectrum taken from the pink square in panel n.

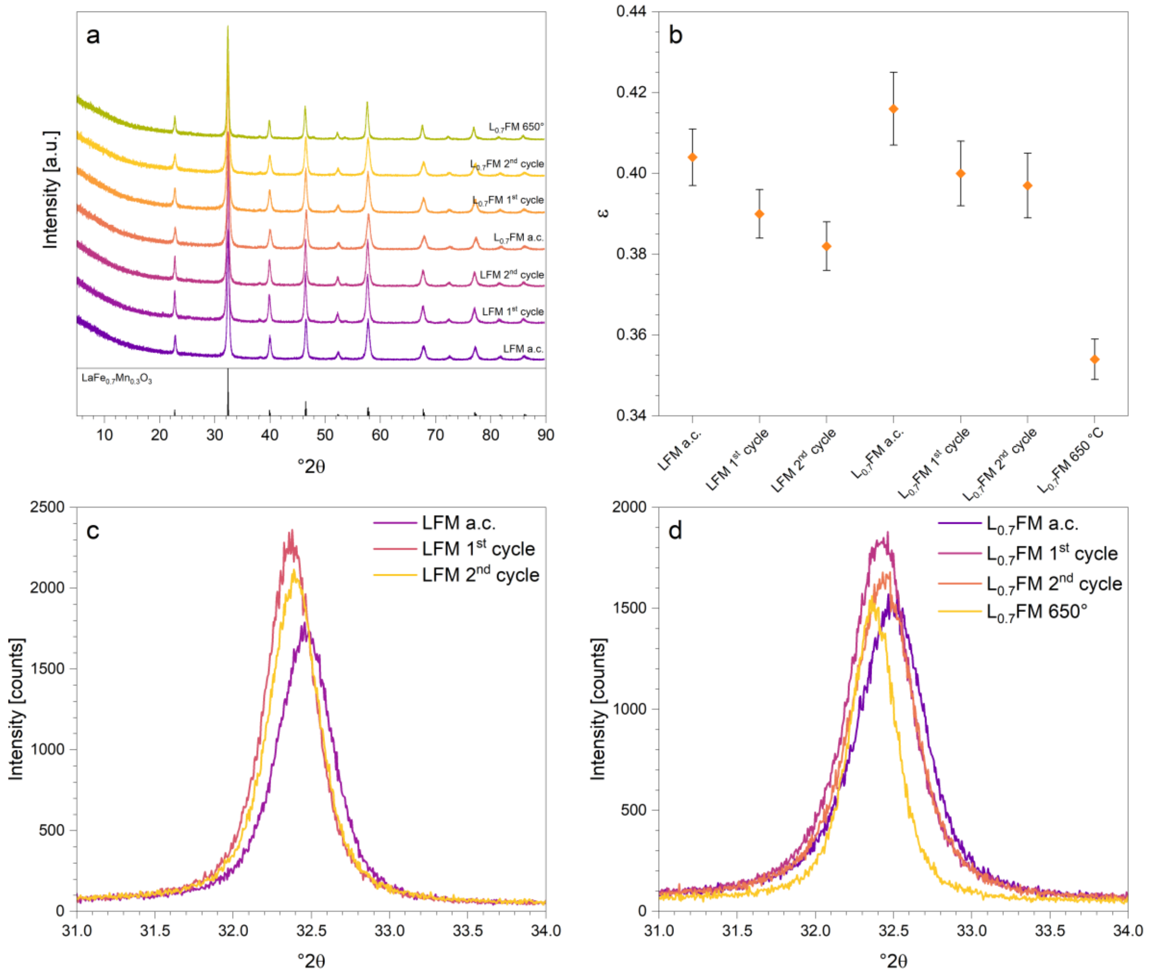

SI Figure 9: (a) Overview of the XRD measurements of LFM and $L_{0.7}$FM samples compared to a LFM reference in the as-calcined state and after selected NO+CO treatments. Panel b: Micro strain ε as calculated from the diffractograms of the LFM and $L_{0.7}$FM samples shown in (a). Panels c and d: Individual (2 0 0) peaks of the LFM and the L0.7FM samples after selected treatments.

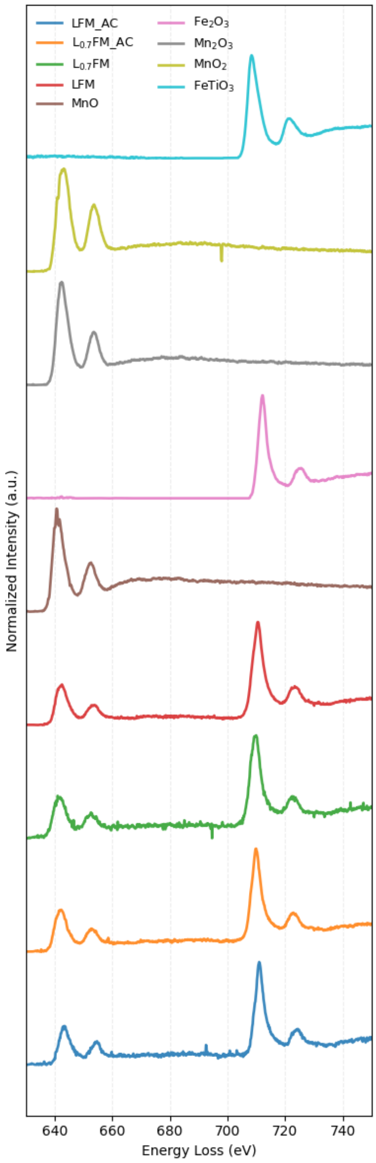

SI Figure 10: Raw spectra of all measured samples including the standard measurements. The spectra are shifted in y-axis for claritiy.

References

[1] Rohamtalei. *Rohamtalei/GPA-Strain-Mapping: Strain Mapping GPA v.1.0.1*; Zenodo, 2025

[2] Mohammadi, A.; Thurner, C. W.; Haug, L.; Bekheet, M. F.; Müller, J. T.; Gurlo, A.; Hejny, C.; Nezhad, P. D. K.; Winkler, D.; Riedel, W.; Penner, S. How defects in lanthanum iron manganite perovskite structures promote the catalytic reduction of NO by CO. Materials Today Chemistry 2024, 35, 101910. DOI: 10.1016/j.mtchem.2024.101910.
